# The Benefits of Promoting Junior Trainees in Vascular Surgery

**DOI:** 10.7759/cureus.50517

**Published:** 2023-12-14

**Authors:** Pierre William McCaughran, Hannah Burton, Mehdi Mohammadi, Matthew J Metcalfe

**Affiliations:** 1 Vascular Surgery, Lister Hospital, Stevenage, GBR; 2 Vascular Surgery, Bedford Hospital, Bedford, GBR

**Keywords:** peripheral vascular surgery, general and vascular surgery, education and training of medical students and doctors (specialist and phd)), higher education medical training, procedure training

## Abstract

Introduction

United Kingdom surgical training consists of a two-year core surgical training (CST) followed by a six-year higher speciality training (ST). There is a significant step up in responsibility and operative skills when transitioning from core to higher training. One-way trainees can bridge this gap is to “act up” to registrar level “CST-R.” The CST “steps up” to the role of ST typically in the latter part of their core training and gains exposure at being the "reg of the week," primary assistant in theatre, managing MDTs, and taking speciality referrals. This can be an excellent training opportunity. This study aims to demonstrate a quantitative improvement in trainee operation as a result of stepping up.

Methods

This study compares the operative experience of one vascular surgery-themed trainee during six months as a CST and six months acting up as a CST-R. The trainee’s eLogbook was searched for all operations between August 3, 2022, and January 31, 2023, and between February 1, 2023, and August 1, 2023. The number of cases performed and the role played in each were analyzed. The number of low complexity cases conducted in each block was used as a baseline to ensure the progression seen was because of increasingly complex ST operating rather than the increase in CST level operating expected throughout CST. An abscess incision and drainage were used as the reference low-complexity case.

Results

The number of cases the trainee performed independently increased from 13% to 25%, while the number where they were simply assisting decreased from 43% to 35%. The number of cases where the trainer remained scrubbed decreased nonsignificantly from 43% to 39%. The number of low-complexity cases performed remained unchanged for each six-month block.

Conclusion

As a CST-R, the trainee played a more prominent operative role in a greater number of cases. The CST-R does require a supportive department and consultant body. It also enables other STs to gain more surgical exposure because of their reduced frequency of being the "reg of the week." If a trainee can remain in a post for two six-month blocks, then there is much to be gained from a formalised acting-up program, and consideration should be given to formally incorporating this into core surgical programs.

## Introduction

Surgical training in the United Kingdom typically comprises two distinct programs: two years of core surgical training (CST), followed by six years of specialist training (ST) as a speciality registrar [1]. During CST, junior surgical trainees () undergo placement in several different, but usually complementary, specialities and are encouraged to develop both their operative and nonoperative skills in a supported environment [2]. They are also obligated to undertake surgical skills courses such as advanced trauma life support (ATLS) [2]. Completion of the Membership of the Royal College of Surgeons (MRCS) examination is an exit requirement for satisfactory CST training completion [2,3].

The advantage of this structure is junior trainees (CSTs) are provided with an extremely well-supported training environment surrounded and trained by their more senior colleagues (STs and consultants). CSTs mostly operate with the direct supervision of a trainer (scrubbed or unscrubbed), which is meant to support the development of good surgical techniques: this is defined by the Intercollegiate Surgical Curriculum Program (ISCP) as Level II supervision (able and trusted to act with direct supervision) [2]. As a result, it is assumed that once a trainee reaches the speciality training (ST3) level, they will be competent to act and operate with an element of indirect Level III supervision (defined by ISCP as able and trusted to act with indirect supervision) [2]. This is assessed from their final multi-consultant report (MCR) at the end of CST, where their level of competence and independence is formally signed off [2].

This structure heavily relies on proactive trainers in the theatre environment. Time pressures, individual attitudes, and the number of trainees competing for operating opportunities among other factors including maintaining compliance with the European Work Time Directive can contribute to the quality of training received [4]. The expectation of CSTs in theatre in terms of their operative ability and needs is often different from that of STs [2,5]. Given current NHS pressures, there is often the request for the CSTs (and less so of STs) to leave theatre and address nonoperative surgical issues in the hospital, which can come at the expense of their theatre exposure [6,7]. In busy periods such as winter or surrounding ongoing strike action, this may result in inadequate access to operative training opportunities [8].

One way in which core trainees (CT) throughout the NHS have traditionally tried to bridge the gap between core and ST is the concept of “acting up” [9]. The CST ‘steps up’ to the role of ST, typically in the latter part of their core training, when the opportunity arises. This gives them exposure to ST clinical work in preparation for their official ST3 year, which often follows [9,10]. Whilst this can be highly beneficial for training, it requires the support of all senior colleagues in understanding that more support will be necessary for the CST-registrar (CST-R). If this is not available, then acting up risks the CST-R feeling overwhelmed and burnt out, struggling to handle the increased responsibilities [11]. This can lead to errors with significant medicolegal consequences and negatively impact their confidence both inside and outside of the operating theatre [12].

The progression from practising under direct to indirect supervision is a large and potentially daunting one. There is a greater emphasis on decision-making, which is particularly evident in acute situations. Given the hierarchical nature of the surgery barriers for a CST to escalate to their ST are much lower than those for an ST to escalate to their consultant [13]. Thus, escalating from a CST-R to a consultant poses the greatest challenge, and a supportive and unintimidating consultant body is essential.

It is relatively difficult to quantitively compare the nonoperative experience of trainees before and after acting up. Operative experience is much easier to quantitively compare as all UK core surgical trainees are required to keep a logbook of cases participated in [2]. This includes coding all procedures according to the degree of involvement in the Surgical eLogbook. The options are assisting (A), supervised trainer scrubbed (STS), supervised trainer unscrubbed but in theatre (STU), performed (P), and training a trainee (T) [10]. In “A,” the trainee performs less than 50% of the operation; in “STS,” they perform over 50%; and in “STU,” “P,” and “T,” they perform the whole procedure with or without the trainer present [10].

We compare the operative experience of a vascular surgery-themed trainee during six months as a core surgical trainee and six months of acting up as a CST-R.

## Materials and methods

A retrospective analysis of the Intercollegiate Surgical Curriculum Program (ISCP) eLogbook was performed. The inclusion criteria were all operations performed by one vascular surgery-themed trainee within a regional vascular hub between August 3, 2022, and January 31, 2023, and all operations performed between February 1, 2023, and August 1, 2023. This corresponds to the full CST year one. The first six-month block corresponded to time spent as a true core surgical trainee (year one) and the second six-month block acting as a registrar (CST-R). Noncoded operations or operations with any role other than (A, STS, STU, P, or T) were excluded. Volume and variation in the complexity of caseload populating operating lists throughout the year were taken to have remained consistent.

Data were collected, and graphs were generated using Microsoft Excel. For each case, data were collected on the role played (A, STS, STU, P, T), the six-month period during which the case was performed, and whether the procedure corresponded to a vascular index procedure (amputation/debridement, vascular access procedure, varicose vein surgery) as defined by the ISCP Vascular Surgery Curriculum [5]. The proportion of cases logged at each level of involvement was compared between the two six-month blocks. Statistical analyses were conducted in Stata (version 18.0; StataCorp LLC, College Station, TX). Pearson’s chi-squared test was used to test the null hypothesis that there was no difference between the proportion of cases performed at each level during each six-month block to the 0.05 significance level.

The number of low-difficulty cases where the trainee was the sole operator (cases logged as STU, P, or T) in each block was used as a baseline to ensure the progression seen was because of increasingly complex ST operating rather than the increased volume in CST level operating expected throughout CST. An abscess incision and drainage were used as the reference low-difficulty case. This was selected as within the Trust, CSTs routinely perform abscess incisions and drainages as the sole operator.

The number of days the trainee was within the hospital and thus available to operate within each block was also compared to account for any reduced operating from days off because of industrial action.

## Results

The trainee conducted a total of 229 cases overall. One hundred twenty cases were performed in the first six-month block as a CST year one, and 109 cases in the second six-month block as a CST-R. Within the first six-month block, 89.5 days were spent in the hospital with the potential to operate, and, in the second, 77.5. This reduction was largely because of NHS industrial action. The 9.2% reduction in total cases performed corresponds to a 13.4% reduction in days spent in the hospital.

As a CST-R, the trainee played a more prominent operative role in a greater number of cases. The number of cases the trainee performed as the sole operating surgeon (STU, P, or T) demonstrated a relative increase of 100% from 16 (13%) to 28 (26%) cases (p=0.018). The number where they were simply assisting demonstrated a relative decrease of 19% from 52 (43%) to 38 (35%) cases (p=0.19). The number of cases where the trainer remained scrubbed also demonstrated a relative decrease of 9% from 52 (43%) to 43 (39%) cases (p=0.55). The number of low-difficulty cases performed independently remained unchanged at 11 for each six-month block.

A total of 91 index procedures were conducted. Forty-nine of these as a CST year one and 43 as a CST-R. The number of procedures recorded as A demonstrated a relative decrease of 33% from 20 (41%) to 12 (28%) cases (p=0.195). Those recorded as STS demonstrated a relative decrease of 12% from 28 (57%) to 22 (51%) cases (p=0.56). The number of cases performed as sole operating surgeons (STU, P, or T) demonstrated a relative increase of 950% from one (2%) to nine (21%) cases (p=0.004). 

**Figure 1:**
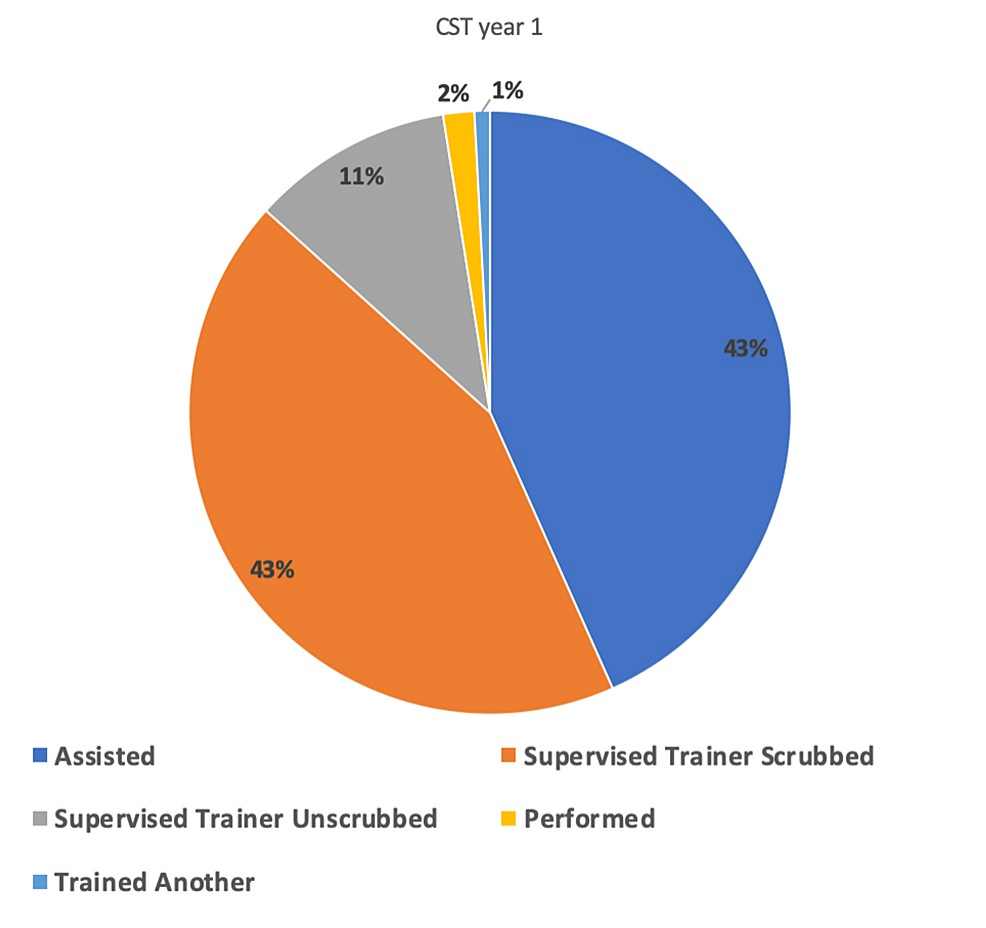
Pie chart depicting the number of cases and the roles played as a CST year one from August 3, 2022, to January 31, 2023.

**Figure 2:**
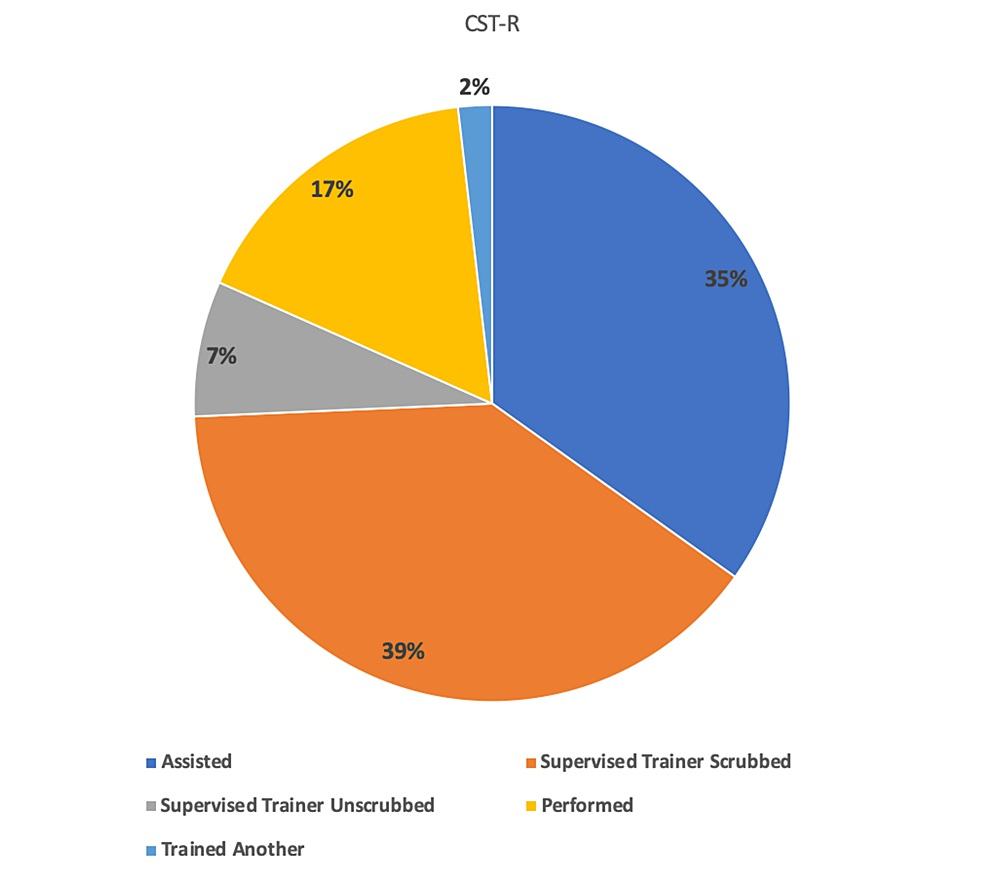
Pie chart depicting the number of cases and the roles played as a CST-R from February 1, 2023, to August 1, 2023.

**Figure 3:**
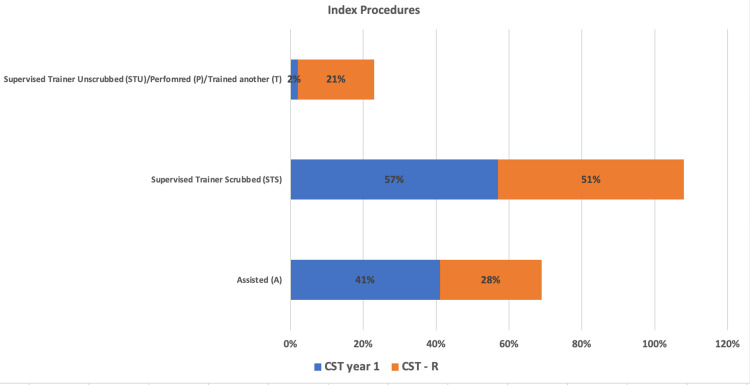
Bar chart demonstrating the total number of index procedures (amputations/debridements, vascular access procedures, and varicose vein procedures) and the roles played as a CST year one from August 3, 2022, to January 31, 2023, and as a CST-R from February 1, 2023, to August 1, 2023.

The total number of cases along with the the breakdown of roles (A, STS, STU, P, and T) for the first- and second six-month blocks are shown in Table 1. The number of index procedures and the breakdown of roles for the first and second six months are shown in Table 2. The percentage breakdown of roles played for all cases for the first six-month block is shown in Figure 1. The percentage breakdown of roles played for all cases for the second six-month block is shown in Figure 2. The percentage breakdown of roles for index procedures for the first and second six-month blocks is demonstrated in Figure 3.

**Table 1:** Total number of cases and the roles played as a CST year one from August 3, 2022, to January 31, 2023, and as a CST-R from February 1, 2023, to August 1, 2023.

Total cases	Assisted (A)	Supervised trainer scrubbed (STS)	Supervised trainer unscrubbed (STU)	Performed (P)	Trained another (T)	Total
CST year 1	52	52	13	2	1	120
CST-R	38	43	8	18	2	109

**Table 2:** Total number of index cases (amputations/debridements, vascular access procedures, and varicose vein procedures) and roles played as a CST year one from 03/08/2022-31/01/2023 and as a CST-R from 01/02/2023-01/08/2023.

Index procedures	Assisted (A)	Supervised Trainer Scrubbed (STS)	Supervised Trainer Unscrubbed (STU)	Performed (P)	Trained another (T)	Total
CST year 1	20	28	1	0	0	49
CST - R	12	22	5	4	0	43

## Discussion

There is a clear trend evident in the results. As a CST-R, the trainee had played a more significant role in a greater number of cases. The number of cases where they were operating independently (STU, P, or T) went from 13% to 26% of the total cases performed within the six-month block, a statistically significant relative increase of 100%. The number of independent low-difficulty cases remained unchanged between the two blocks. This indicates the CST-R continuing to perform typical CST level cases whilst adding more complex ST level operating to their repertoire. That the number of index procedures conducted (STU, P, or T) demonstrated a significant increase from 2% to 21% supports this within the caveat of the small sample size. Although not of statistical significance, the decrease in the number of index procedures recorded as A may indicate that CST-R plays a more significant role in vascular index procedures both operating alone and being trained. The relatively small decrease in overall cases recorded as A or STS demonstrates the increased independent operating is unlikely to have greatly detracted from the CST-R's one-to-one operating with a scrubbed trainer or their exposure to the most complex cases as an assistant: both important parts of surgical training. Notably, the P value for the change in both A and STS cases overall and for index procedures was >0.05. Therefore, it is only possible to attribute statistical significance to the changes in operation observed in this study where the CST-R was the sole operator.

The longer a CST remains in a department, the more their abilities and skill level will be clear to their trainers. This tends to result in increasing levels of trust and independence with time. However, it is unusual for CSTs to be left to operate independently without a trainer in the operating theatre as this corresponds to a level III on the ISCP Multi Consultant Report, and it is an exit requirement of the program, not expected until the last placement of CST [3]. Operating without a trainer directly available to guide and instruct forces the development of operative decision-making and reasoning under pressure [14]. This is a very important skill that is expected of STs, and it is a source of significant stress amongst junior registrars when they begin their higher training [11]. This work shows that acting up provides opportunities to develop these skills in a more supportive and controlled environment, which could help in reducing the anxiety and stress experienced by trainees on beginning higher training. 

Expectations regarding the complexity of operations performed also change between CST and ST [2,5]. The CST curriculum requires competence to be achieved (defined as level III trusted to act with indirect supervision) in six operative procedures: scrubbing, gloving, and gowning, preparing an aseptic field, injecting local anesthetic, and incising and closing a wound [2]. As they progress in our Trust, CSTs may also be expected to perform “simple” procedures such as incision and drainage of an abscess unsupervised. STs are expected to perform more complex procedures and the ISCP vascular curriculum requires "index procedures" to be signed off as level IV (defined as able and trusted to act at the level expected of a day-one consultant) as trainees progress [5]. The step up as a CST-R offers exposure to operating at a higher level provided that the CST-R is competent to perform the more complex procedures. The experience of ST-level operating is extremely good preparation for higher training. For the trainee in this study, all cases performed “STU,” “P,” or “T” were discussed preoperatively in a step-by-step manner and then again postoperatively with a consultant. This allowed for reflection on difficulties encountered and how the trainee addressed them. Examples of cases performed in this way include above and below-knee amputations, forefoot and toe amputations, major wound debridements, peritoneal dialysis catheter insertions and removals, arteriovenous fistula formations, and varicose vein ablations. These are all vascular surgery "index procedures" [5]. No complications arose from any of the procedures undertaken by the trainee “STU,” “P,”, or “T” as a CST-R.

There is also a benefit to other STs in the department in having a CST-R. In our department, STs cover the senior workload in three main areas: the acute Take, the ward, and the operating theatre. The acute take and the ward are covered by a “Reg of the Week” (RoW). The RoW has no scheduled operations during this period to ensure availability for their nonoperative duties, other than emergency vascular operations. The promotion of the CST-R ensured that the other STs did a reduced frequency of RoW and thus a corresponding increase in scheduled operating lists. It also improved opportunities for other STs to take study leave and attend courses because of the reduced frequency of scheduled commitments. 

Despite the numerous positives from the CST-R program, there are nonetheless some potential drawbacks. The relative lack of experience of the CST-R means that senior colleagues must provide more support than they otherwise would for an ST. The promotion of the CST results in one fewer member of junior staff on the ward for STs to delegate to. This may increase the overall workload of the RoW on the ward. The supervising consultant will also need to provide more support for the CST-R during their periods as RoW to ensure all senior ward and acute take duties are carried out safely. Finally, because of a lack of experience, the CST-R will inevitably operate slower than their more experienced colleagues, and this needs to be accounted for when planning lists. 

A buddy system was used for the trainees in this study. Whenever they were RoW and covering the acute take, a senior registrar was scheduled to be available as the first port of call. This enabled minor clinical issues and logistics to be addressed without the need to involve the consultant on call. A similar concept is often used in hyperacute surgical specialties such as neurosurgery or trauma surgery where first-year STs (ST3) are placed on a two-tier rota: the junior registrar is the tier one, and a senior registrar is their tier two; there is then a supervising consultant on call from home [15]. This structure ensures that approachable support is always available for the junior trainee whilst protecting the consultant body from being harassed unnecessarily when on call.

This study has several limitations. These include a reliance on accurate theatre data being entered into the eLogbook. It was assumed that the trainee uploaded 100% of theatre cases accurately. If this study were to be repeated, there is a risk that minor procedures are omitted as the CST-R focuses on more complex procedures as they improve their surgical abilities. 

The second six-month period analyzed for this study corresponded with industrial action on a scale never before seen in the NHS [16]. There was an approximate 12% reduction in elective vascular operating lists at our Trust. Within our department, elective cases are frequently the cases where a CST-R would play a greater role because of the complexity of cases and time allocation. Many routine elective operating lists were canceled, and priority was given to emergencies [17]. Many of the routine elective operations canceled would have been cases where trainees would be operating.

Another major limitation is that this study was only addressed from the perspective of the CST-R. The opinions of both the other STs and the consultant body are not reported. Similarly, the impact of losing an approachable CST on the ward for the foundation year one (F1) doctors was not reported.

The final caveat relates to the structure of CST rotations in the UK. Most two-year programs are made up of four six-month rotations through different specialities [1]. For a trainee to achieve the experience and level of trust to act up within a speciality they need to be in post for a significant length of time. This ideally requires two six-month rotations within the same speciality and the same department. It is interesting to note that these changes were observed within the CT1 year rather than the traditional CT2 year and the trainee in question had undertaken a year of surgical training in a different speciality before starting CST.

## Conclusions

Acting up can be an invaluable way for core surgical trainees to develop their operative and nonoperative skills. It provides greater independence and endorses the development of clinical reasoning and decision-making. It enables the CST to practice their leadership skills and more senior-level communication skills. It also offers significantly more operative opportunities to better prepare a trainee for their higher specialty training. It nonetheless requires a supportive department and consultant body, and, if this is not ensured, there is potential for junior trainees to be rapidly overwhelmed with ensuing personal and medicolegal ramifications. The structure of CST in the UK with its frequent rotations between specialties and departments is not always conducive to creating this environment. If a trainee can remain in post for two six-month blocks then there is much to be gained from a formalized acting-up program and consideration should be given to designing core surgical rotations with this in mind. In our Trust, this opportunity is also available in urology and is thought to be successful.

## References

[1] (2023). Royal college of surgeons: surgery entry requirements and training. Surgeons.

[2] (2023). Intercollegiate surgical curriculum program. https://www.iscp.ac.uk/iscp/curriculum/core-surgical-training-curriculum/5-programme-of-assessment/.

[3] (2023). Joint committee on surgical training: core surgical training. Joint Committee on Surgical Training.

[4] Datta ST, Davies SJ (2014). Training for the future NHS: training junior doctors in the United Kingdom within the 48-hour European working time directive. BMC Med Educ.

[5] https://www.iscp.ac.uk/media/1113/vascular-surgery-curriculum-aug-2021-approved-oct-20.pdf ISCP Vascular Surgery Curriculum 4 August 2021 (2023). Vascular surgery curriculum: Intercollegiate surgical curriculum program. https://www.iscp.ac.uk/media/1113/vascular-surgery-curriculum-aug-2021-approved-oct-20.pdf.

[6] (2023). General Medical Council: national training surveys result. https://www.gmc-uk.org/-/media/documents/2017_national_training_surveys_summary_report_initial_results_on_doctors_training_progression.pdf_71003116.pdf.

[7] (2023). JCST trainee survey annual report - 2020/21 and 2021/22. https://www.jcst.org/quality-assurance/trainee-survey/.

[8] (2023). Health Education England: supporting winter pressures safely through managed education and training programmes. https://www.hee.nhs.uk/sites/default/files/documents/HEE%20winter%20pressures%20guidance%20FINAL.pdf.

[9] (2023). Guidance on core medical trainees acting up as a medical registrar. https://www.jrcptb.org.uk/sites/default/files/Guidance%20on%20Core%20Medical%20Trainees%20acting%20up%20as%20a%20Medical%20Registrar.pdf.

[10] (2023). Intercollegiate surgical curriculum program logbook. https://www.iscp.ac.uk/static/help/operations.pdf.

[11] Robinson DB, Luton O, Mellor K (2021). Trainee perspective of the causes of stress and burnout in surgical training: a qualitative study from Wales. BMJ Open.

[12] Zhou AY, Hann M, Panagioti M (2022). Exploring associations between stressors and burnout in trainee doctors during the COVID-19 pandemic in the UK. Acad Psychiatry.

[13] Cosby KS, Croskerry P (2004). Profiles in patient safety: authority gradients in medical error. Acad Emerg Med.

[14] Flin R, Youngson G, Yule S (2007). How do surgeons make intraoperative decisions?. Qual Saf Health Care.

[15] (2023). UK Neurosurgery Workforce Report 2020. https://www.sbns.org.uk/index.php/policies-and-publications/.

[16] (2023). NHS industrial action: the impact on patients, staff and performance. https://nhsproviders.org/media/696855/nhs-providers-briefing-nhs-industrial-action-the-impact-on-patients-staff-and-performance-july-2023-members.pdf.

[17] (2023). Information for the public on industrial action. https://www.england.nhs.uk/long-read/information-for-the-public-on-industrial-action/.

